# Don’t be late! Timely identification of cognitive impairment in people with multiple sclerosis: a study protocol

**DOI:** 10.1186/s12883-023-03495-x

**Published:** 2024-01-13

**Authors:** Pauline T. Waskowiak, Brigit A. de Jong, Bernard M. J. Uitdehaag, Shalina R. D. Saddal, Jip Aarts, Aïda A. M. Roovers, Pim van Oirschot, Vincent de Groot, Frederieke G. Schaafsma, Karin van der Hiele, Marit F. L. Ruitenberg, Menno M. Schoonheim, Guy A. M. Widdershoven, Sabina van der Veen, Esther C. F. Schippers, Martin Klein, Hanneke E. Hulst, Don’t be late! Consortium, Casper E. P. van Munster, Renske G. Wieberdink, Jolijn J. Kragt, Judith Schouten, Erwin L. J. Hoogervorst, Paul A. D. Bouma, Floris G. C. M. De Kleermaeker, Meike Holleman, Sofie Geurts, Christaan de Brabander, Nynke F. Kalkers, Bram A. J. den Teuling, Jos Vermeer, Chris C. Schouten, Gerard J. Stege, Thijs van‘t Hullenaar

**Affiliations:** 1 MS Center Amsterdam, Medical Psychology, Vrije Universiteit Amsterdam, Amsterdam Neuroscience, Amsterdam UMC Location VUmc, De Boelelaan 1118 Amsterdam, The Netherlands; 2 MS Center Amsterdam, Neurology, Vrije Universiteit Amsterdam, Amsterdam Neuroscience, Amsterdam UMC Location VUmc, Amsterdam, The Netherlands; 3 MS Center Amsterdam, Public and Occupational Health, Vrije Universiteit Amsterdam, Amsterdam UMC Location VUmc, Amsterdam, The Netherlands; 4 Health, Medical and Neuropsychology Unit, Institute of Psychology, Faculty of Social Sciences, Leiden University, Leiden, The Netherlands; 5 Leiden Institute for Brain and Cognition, Leiden, The Netherlands; 6 MS Center Amsterdam, Anatomy and Neurosciences, Vrije Universiteit Amsterdam, Amsterdam Neuroscience, Amsterdam UMC Location VUmc, Amsterdam, The Netherlands; 7 Sherpa B.V., Nijmegen, The Netherlands; 8 MS Center Amsterdam, Rehabilitation Medicine, Vrije Universiteit Amsterdam, Amsterdam UMC Location VUmc, Amsterdam, The Netherlands; 9 Ethics, Law & Medical Humanities, Vrije Universiteit Amsterdam, Amsterdam UMC Location VUmc, Amsterdam, The Netherlands

**Keywords:** Multiple sclerosis, Cognitive impairment, Neuropsychology, Digital screening, Innovation, Health-related quality of life

## Abstract

**Background:**

Cognitive impairment occurs in up to 65% of people with multiple sclerosis (PwMS), negatively affecting daily functioning and health-related quality of life. In general, neuropsychological testing is not part of standard MS-care due to insufficient time and trained personnel. Consequently, a baseline assessment of cognitive functioning is often lacking, hampering early identification of cognitive decline and change within a person over time. To assess cognitive functioning in PwMS in a time-efficient manner, a BICAMS-based self-explanatory digital screening tool called the Multiple Screener^©^, has recently been developed. The aim of the current study is to validate the Multiple Screener^©^ in a representative sample of PwMS in the Netherlands. Additionally, we aim to investigate how cognitive functioning is related to psychological factors, and both work and societal participation.

**Methods:**

In this cross-sectional multicentre study, 750 PwMS (aged 18–67 years) are included. To obtain a representative sample, PwMS are recruited via 12 hospitals across the Netherlands. They undergo assessment with the Minimal Assessment of Cognitive Functioning in MS (MACFIMS; reference-standard) and the Multiple Screener^©^. Sensitivity, specificity, and predictive values for identifying (mild) cognitive impairment are determined in a subset of 300 participants. In a second step, the identified cut-off values are tested in an independent subset of at least 150 PwMS. Moreover, test–retest reliability for the Multiple Screener^©^ is determined in 30 PwMS. Information on psychological and work-related factors is assessed with questionnaires.

**Discussion:**

Validating the Multiple Screener^©^ in PwMS and investigating cognition and its determinants will further facilitate early identification and adequate monitoring of cognitive decline in PwMS.

## Background

Multiple sclerosis (MS) is a chronic inflammatory disease of the central nervous system characterized by demyelination and neurodegeneration [1]. In addition to physical limitations, 43–65% of people with MS (PwMS) develop cognitive symptoms that may severely affect daily life functioning and consequently health-related quality of life [2–4]. The most commonly and earliest affected cognitive domains are information processing speed, verbal memory, and visuospatial memory [3, 5].

The impact of cognitive impairment on daily life functioning can be significant, especially since most PwMS are relatively young at disease onset [3]. As such, cognitive impairment is one of the main reasons for unemployment in MS [6, 7]. About 43% of PwMS become unemployed within three years of diagnosis due to fatigue and physical impairment, but also cognitive impairment [8–10]. This early unemployment has a large impact on PwMS, their families, and on society in general [11]. However, by the time PwMS with self-perceived cognitive problems approach health care professionals, their cognitive deficits are often already advanced and potentially more difficult to treat, suggesting that early intervention might be promising [12, 13].

The need for early intervention is emphasized by a recent study showing that successful response to cognitive rehabilitation depends on the status of the brain’s functional network before the intervention [14]. PwMS with a functional connectivity that is more like that of healthy controls were able to benefit from a cognitive rehabilitation program (i.e., these participants significantly improved on neuropsychological tests and had better self-perceived cognitive functioning). However, those PwMS with a less efficient brain network at baseline (indicative of more MS-related pathology) were non-responsive, suggesting the existence of a small window of opportunity for intervention early in the development of the disease [14]. Additionally, other studies show that less MS-related brain damage (e.g., higher grey matter volume) are linked to better cognitive rehabilitation outcomes [15–17]. Therefore, it is crucial to identify PwMS at the earliest stages of cognitive impairment to allow for intervention when it is most effective to improve cognitive functioning.

Recent international recommendations for measuring and monitoring cognitive functioning in PwMS propose a baseline cognitive screening and annual follow-up [18]. However, in contrast to these recommendations, neuropsychological testing is not part of standard MS-care in current clinical practice in the Netherlands (and several other countries) [19]. Consequently, a good reference assessment of baseline cognitive performance is often lacking, hampering the detection of the first subtle changes in cognition. Detecting these early changes is particularly difficult when PwMS already experience difficulties in daily life functioning but still perform (above) average on neuropsychological assessments [3]. The main reason for not following the international recommendations is the lack of time and specialized personnel to assess cognitive functioning [19]. As digital assessment tools may lower the threshold for systematic assessment of cognitive functioning in PwMS, we recently developed a self-explanatory, time-efficient digital screening tool, the Multiple Screener^©^ [20].

The Multiple Screener^©^ consists of an adjusted version of the validated and recommended BICAMS (Brief International Cognitive Assessment for MS) paper-and-pencil assessment [21] and takes 15 min to complete. It assesses the most frequently impaired cognitive domains in MS: information processing speed via the Symbol Digit Modalities Test (SDMT [22]), verbal learning and memory via the Dutch version of California Verbal Learning Test Second Edition (CVLT-II [23–25]), and visuospatial learning and memory via the Spatial Recall Test (SPART [26]) [20]. In addition, the Multiple Screener^©^ also includes questionnaires on depression and anxiety [27], fatigue [28] and self-perceived cognitive symptoms [29], taking into account psychological factors when screening for cognitive deficits in MS. The main advantages of the Multiple Screener^©^ are that it does not require specialized personnel for administration (i.e., PwMS can perform the tests on their own), has automated scoring, and is time-efficient. The Multiple Screener^©^ has been tested in 236 healthy controls and normative data are available [20]. In healthy controls, the correlations between the Multiple Screener^©^ and the paper-and-pencil versions of the neuropsychological tests have been shown to be good to excellent [20]. However, a next essential step before the Multiple Screener^©^ can be used in clinical practice is to investigate its diagnostic accuracy especially in identifying PwMS with mild cognitive impairment according to a reference standard (the Minimal Assessment of Cognitive Function in Multiple Sclerosis, MACFIMS [30]), allowing for timely identification.

### Objectives

As part of a larger research project (i.e., the *Don’t be late! study,* see Table 1) the primary objective of this study is to determine diagnostic accuracy of the Multiple Screener^©^ in a representative Dutch sample of PwMS. Specifically, we aim to determine how well the Multiple Screener^©^ can differentiate between PwMS with no cognitive impairment, mild cognitive impairment, and cognitive impairment according to the reference-standard (MACFIMS [30]). In a second step we aim to confirm the observed diagnostic accuracy of the Multiple Screener^©^ in differentiating between PwMS with no cognitive impairment, mild cognitive impairment, and cognitive impairment in an independent subset of PwMS. When reporting on the diagnostic accuracy of the Multiple Screener^©^, the Standards for Reporting Diagnostic Accuracy guidelines from the Equator-Network (STARD 15, [31]) will be followed.

**Table 1:** Don’t be late study!

The *Don’t be late!* study consists of three work packages (WPs) with the overarching goal to postpone cognitive decline and prevent early unemployment in PwMS. While WP1 focuses on early identification of cognitive impairment, WP2 will investigate the effectiveness of two personalized preventative interventions on health-related quality of life in PwMS. A selection of participants that are included in WP1 (i.e., participants with mild cognitive impairment [who are therefore expected to still benefit from the interventions] and working for at least 12 h a week), will be invited to partake in WP2. Finally, WP3 aims to foster the implementation of these interventions according to patients needs and by including relevant stakeholders	

The study has the following secondary objectives:

1. To determine the test–retest reliability of the Multiple Screener^©^;
2. To determine how cognitive, psychological, work-related, and health-related quality of life outcomes are related.

## Methods

### Design and setting

The present study is a cross-sectional multicentre study in which a representative sample of 750 PwMS will be included. In the 12 participating Dutch hospitals, demographical and medical information will be collected, and cognitive functioning of PwMS will be assessed with both the reference standard (MACFIMS) and the Multiple Screener^©^. In line with international validation guidelines [32], the assessment of the Multiple Screener^©^ will be repeated within 3 weeks after the hospital visit in a small subset of participants (*N* = 30) in order to determine test–retest reliability. Finally, all participants will fill in several online questionnaires at home.

### Participants

#### Recruitment and consent

We aim to recruit a representative sample of PwMS in the Netherlands that visit the neurologist in light of their MS. We will include PwMS with a variety in MS types (relapsing remitting, secondary progressive (85% of the population) and primary progressive (15%)), disease duration and age. All participating hospitals are asked to provide a patient information letter for a set period of time to all PwMS that visit the outpatient clinic, independent of cognitive status, employment status, disease status and meeting the in- and exclusion criteria. Contact details of PwMS that give permission to be approached about participation are shared with the researchers from the Amsterdam UMC, Vrije Universiteit Amsterdam. After at least one week, they contact the potential participant to provide additional information if requested (or refer to an independent physician) and to ask whether they would like to participate in the study. When PwMS decide to participate, the researcher will screen the subjects for eligibility via telephone (see below for inclusion and exclusion criteria) such that an unnecessary hospital visit will be avoided when a subject is not eligible. In case of a positive screening outcome, a visit for the assessment will be scheduled at which written informed consent will be obtained.

#### Inclusion criteria

To be eligible to participate in this study, people must fulfil the following criteria: a confirmed MS diagnosis according to the McDonald 2017 criteria [33], age between 18 and 67 years, no changes in disease modifying therapy within the last 3 months, and no relapse or steroid treatment six weeks prior to the study visit.

#### Exclusion criteria

Participants will be excluded from participation in this study if they have other neurological or psychiatric comorbidities that can potentially influence cognitive functioning, a current or history of drug or alcohol abuse, have insufficient vison or hearing, or are unable to speak or read Dutch**.** The reasons for excluding participants from the current study will be documented.

#### Ethical approval

The study will be conducted according to the principles of the Declaration of Helsinki (2013) and in accordance with the Dutch Medical Research Involving Human Subjects Act (WMO). The Medical Ethical Committee (METC) of the Amsterdam UMC, Vrije Universiteit Amsterdam has approved this study (METC 2021.0707) on 4 May 2022.

### Measures and procedures

#### Demographic and clinical characteristics

During the assessment at the participating hospitals, information on demographical and clinical characteristics will be collected from participants and their medical file. The following characteristics will be collected: age in years, sex, educational level (Dutch Verhage scale), work status, date of diagnosis, MS subtype, MS severity assessed with the telephone version of the Expanded Disability Status Scale (EDSS) [34], medication usage, medical history, and comorbidities.

#### Neuropsychological assessment

Participants will undergo an extensive neuropsychological assessment (120–150 min) at the location of the participating site. The assessment consists of the Multiple Screener^©^, the MACFIMS test battery [30], a social cognition test, performance validity tests and an assessment of awareness of cognitive functioning. Parallel versions will be used for the tests that are overlapping between the MACFIMS and Multiple Screener^©^ and the order of administration will be counterbalanced to minimize learning effects and influence of fatigue.

##### Multiple Screener^©^

**Fig. 1:**
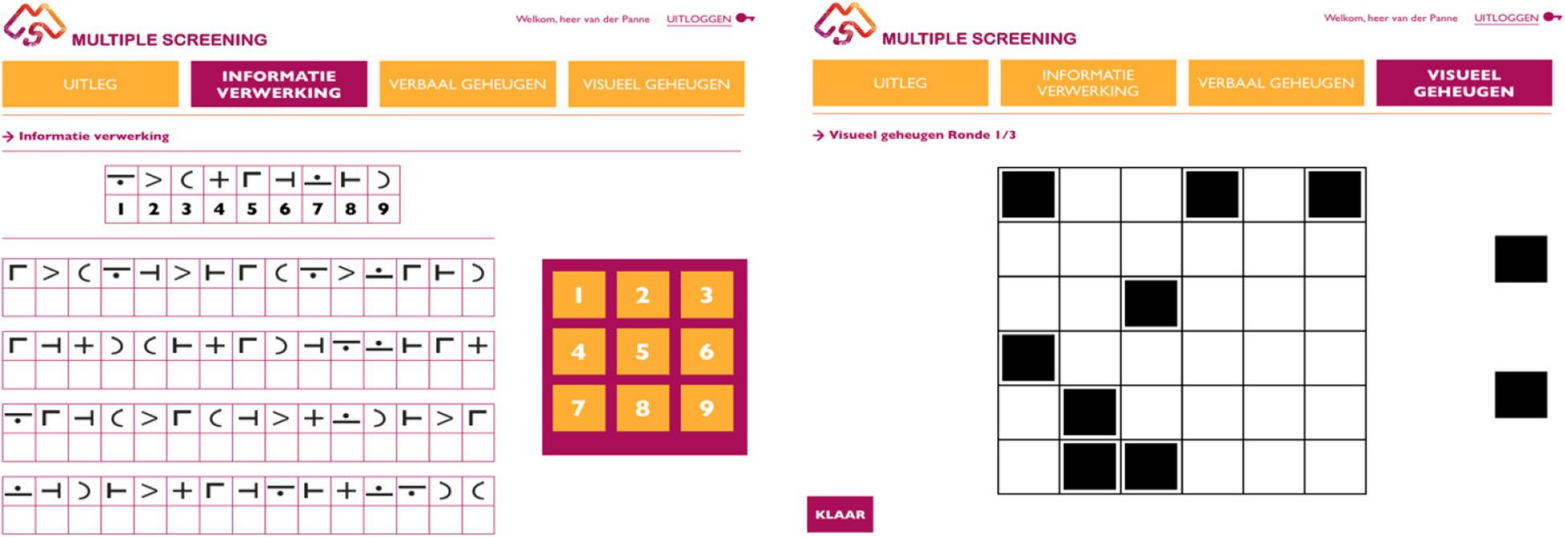
The digital version of the Digit Modalities Test (SDMT) (left) and the Spatial Recall Test (SPART) (right) in the Multiple Screener^©^ application. The Dutch version of the California Verbal Learning Test–second edition (CVLT-II) is not depicted as this test has an auditory format

The Multiple Screener^©^ is a digital tool aiming to assess cognitive functioning in PwMS. It is a digital, self-explanatory version of the validated and recommended BICAMS [21] and takes 15 min to complete. It includes the following three tests:

- Digital version of the CVLT-II [23–25]: Verbal learning and memory. The ability to learn 16 auditory presented semantically related words is examined over five trials. After each trial participants are asked to type the remembered words (direct recall). The total number of the correctly remembered words is calculated.
- Digital version of the SDMT [22]: Processing speed and working memory. Nine pairs of digits and symbols are visually presented. Participants are asked to type the numbers associated with the paired symbols as fast as possible. The total number of correct answers within 90 s is calculated.
- Digital version of the SPART [26]: Visuospatial memory. A 6 × 6 grid with 10 black checkers is displayed three times for ten seconds. After each time, an empty grid is displayed with ten black checkers next to it. Participants must swipe the black checkers to the correct places in the empty grid to match what they observed. The total number of correctly placed checkers is calculated. See Fig. 1 for an illustration of the SDMT and the SPART.

The software of the Multiple Screener^©^ is produced by the manufacturer Sherpa B.V. In accordance with the legislation of the Medical Device Directive, the software is qualified as a medical device, classified in risk class I (low risk), reported to FARMATEC-CIBG-VWS, and CE-certified by the manufacturer.

A subset of participants (*n* = 30) will be invited to return to the hospital within 3 weeks after the initial assessment to complete The Multiple Screener^©^ for a second time to determine the test–retest reliability.

##### MACFIMS

The MACFIMS is an internationally renowned and well-validated, 90-min, paper-and-pencil test battery that is commonly used to determine cognitive impairment in MS. It consists of tests for verbal and visuospatial learning and memory and information processing speed (cf. the Multiple Screener^©^) and in addition tests for language and working memory, visuospatial orientation, and executive functioning [30]. The tests and corresponding cognitive domain(s) are summarized in Table 2.

**Table 2:** Minimal assessment of cognitive functioning in MS test battery

Test	Cognitive domain(s)
Dutch Version of the California Verbal Learning Test, Second Edition (CVLT-II) [23–25]	Verbal learning and memory
Brief Visuospatial Memory Test-Revised (BVMT-R) [35]	Visuospatial learning and memory
Symbol Digit Modalities Test (SDMT) [22]	Information processing speed
Paced Auditory Serial Addition Test (PASAT) [36]	Information processing speed
Controlled Oral Word Association Test (COWAT) [37]	Language and working memory
Judgment of Line Orientation Test (JLO) [37]	Visuospatial orientation
Delis-Kaplan Executive Function System sorting test (D-KEFS) [38]	Executive functioning

##### Performance validity

The Amsterdam Short Term Memory Test (ASTM) [39] will be used to assess performance validity in all participants. In case the ASTM indicates underperformance (cut-off of ≤ 84; [40]) the Rey 15-item Test (higher specificity compared to the ASTM) [41] will additionally be performed.

##### Social cognition

Social cognition and in particular affective theory of mind (i.e., the ability to recognise the thought or feelings of others) will be measured with the revised version of the Reading the Mind in the Eyes Test [42].

##### Awareness of cognitive functioning

Finally, to assess (online) awareness of global cognitive functioning, a subset of the participants (*N* = 200) will be asked to estimate their own performance immediately before and after completion of the MACFIMS battery. More specifically, they will be asked to estimate what percentile score they believe that they would receive for the overall test battery if compared with a randomly selected demographically matched peer group. A normal distribution including brief explanations of percentiles scores (inspired by Rothlind et al. [43]) will serve as a visual aid for participants.

#### Questionnaires

To reduce the burden on the day in the hospital, participants will fill out several online questionnaires at home (for an overview of the questionnaires see Table 3). Participants will be asked to complete the questionnaires within one week after the hospital visit to ensure that the collected data most closely resembles the status of the participant during the hospital visit. The researcher will send reminders if the questionnaires have not been returned.

**Table 3:** Questionnaires on MS-related and psychological factors, work and societal participation

Domain	Measure(s)
Health-related quality of life	MOS 36-Item Short Form (SF-36) [44]
Physical and psychological impact of MS	Multiple Sclerosis Impact Scale (MSIS-29) [45]
Self-perceived cognitive functioning	Multiple Sclerosis Neuropsychological Screening Questionnaire (MSNQ) [29]
Anxiety and depression	Hospital Anxiety and Depression Scale (HADS) [27]
Fatigue	Modified Fatigue Impact Scale (MFIS) [28]
Sleep	Athens Insomnia Scale (AIS) [46, 47]
Resilience	Connor Davidson Resilience Scale (CD-RISC 25) [48]
Mastery	Pearlin Mastery Scale (PMS) [49]
Personality	NEO Five-Factor Inventory (NEO-FFI) [50–52]
Stressful life events	List of Threatening Events Questionnaire (LTE) [53]
Work functioning & work productivity	Work Productivity and Activity Impairment Questionnaire (WPAI) [54]; Multiple Sclerosis Work Difficulties Questionnaire (MSWDQ-23) [55, 56]; Buffalo Vocational Monitoring Survey (BVMS NL-version) [57]
Lifestyle and social participation	In-house developed Lifestyle Factors Questionnaire: assessing health-related lifestyle factors (e.g., smoking, drinking, weight, and height to calculate BMI, exercise, diet,), social activities and information regarding the living situation of participants

*MS* Multiple Sclerosis, *BMI* Body Mass Index

### Outcomes

#### Primary outcome

The primary outcome measures for the first study objective are sensitivity, specificity, negative and positive predictive value, and the receiver-operating characteristic of the Multiple Screener^©^.

#### Secondary outcomes

A secondary outcome measure is the test–retest reliability (i.e., intraclass correlation coefficients) of the Multiple Screener^©^. Additionally, secondary outcome measures include the relationships between cognitive functioning (as measured with the Multiple Screener^©^ and the MACFIMS test battery [30]) with the following measures (the hypothesized directions of these relationships are summarized in Table 4):

1. *Psychological measures:* self-perceived cognitive functioning [29], awareness of cognitive functioning [43], physical and psychological impact of MS [45], mood [27], fatigue [28], personality traits [50, 52], stressful life events [53], resilience [48] and mastery [49]
2. *Patient-reported health-related quality of life* [44]
3. *Work-related measures:* MS-related work difficulties [55], work productivity and activity impairment [54], negative work events and work accommodations [57]
4. *Health and lifestyle measures:* Physical exercise, smoking, alcohol, diet, sleep, BMI, household composition, and social activities.

**Table 4:** Hypothesized direction of correlations between cognitive scores with health-related quality of life, psychological, and work-related, health and lifestyle measures

Measure	Hypothesized direction
**Health-related quality of life**	
SF-36 [44]	+
**Psychological measures**	
MSIS-29 [45]	-
MSNQ [29]	-
HADS [27]	-
MFIS [28]	-
AIS [46, 47]	-
CD-RISC 25 [48]	+
PMS [49]	NA
NEO-FFI [50–52]	NA
LTE [53]	NA
**Work-related measures**	
WPAI [54]	NA
MSWDQ-23 [55, 56]	-
BVMS NL-version [57]	NA
**Health and lifestyle measures**	
Physical exercise	+
Smoking	-
Alcohol use	-
Diet	NA
Sleep	+
BMI	-
Social activities	+

*NA* Not Applicable, as no hypothesis can be formulated beforehand

*Abbreviations*: *MSIS-29* Multiple Sclerosis Impact Scale, *MSNQ* Multiple Sclerosis Neuropsychological Screening Questionnaire, *HADS* Hospital Anxiety and Depression Scale (HADS), *MFIS* Modified Fatigue Impact Scale, *AIS* Athens Insomnia Scale, *CD-RISC* Connor Davidson Resilience Scale, *PMS* Pearlin Mastery Scale, *NEO-FFI* NEO Five-Factor Inventor, *LTE* List of Threatening Events Questionnaire, *WPAI* Work Productivity and Activity Impairment Questionnaire, *MSWDQ-23* Multiple Sclerosis Work Difficulties Questionnaire, *BVMS NL-version* Buffalo Vocational Monitoring Survey, *BMI* Body Mass Index

### Power calculation

Because the Multiple Screener^©^ is aimed at assessing cognitive decline, especially sensitivity to detect (mild) cognitive impairment should be high, while a relatively lower degree of specificity can be tolerated. Based on accuracy values from the paper–pencil version of the Multiple Screener^©^ (BICAMS) in MS and comparable cognitive screening instruments frequently used in people with Parkinson’s disease, we aim for sensitivity values of at least 0.80 and specificity values of at least 0.70 [58, 59]. As reported by Amato et al. [60] we expect that approximately 50% of PwMS at the outpatient clinics will classify as having no cognitive impairment (i.e., cognitively preserved; CP), 30% will classify as having mild cognitive impairment (MCI), and 20% will classify as having overt cognitive impairment (CI). Based on these prevalence estimations, a minimum sample size of 100 will be required to detect sensitivity values of at least 0.80 with a power of 80% and alpha threshold of 0.05 [61]. In total 198 participants will be required to detect specificity values of at least 0.70 with identical power and significance level. However, a sample size of 300 is recommended to reliably evaluate accuracy values of screening tools [61]. As such, for our primary objective we aim to include 300 PwMS. Moreover, we aim to confirm the accuracy of the Multiple Screener^©^ in an independent sample of at least 150 PwMS (i.e., another subset of our sample).

This study is part of a larger research project and a subset of participants from the current study (i.e., participants with mild cognitive impairment) will be selected for the intervention study of the second work package (see Table 1). Therefore, the overall required sample size (*N* = 750) is based on the power calculation for the intervention study. For additional information, the reader is referred to Aarts et al. [62].

### Statistical analysis

Data will be analysed using R Studio software (at least version 4.2.1; [63]) and IBM SPSS Statistics (at least version 28 [64]). In case of non-normality, data will be presented as median and inter-quartile range and transformed for further analyses if appropriate or non-parametric tests will be applied. Participants with missing data and outliers will be excluded for that particular analysis. A *p*-value of 0.05 will be considered as statistically significant for all analyses.

#### Primary study parameters

For our primary objective we will determine sensitivity, specificity, positive and negative predictive values of the Multiple Screener^©^ as compared to the MACFIMS. Based on previous definitions for cognitive impairment among PwMS [65], participants will be divided into three subgroups depending on their severity of cognitive impairment. Participants scoring at least 2 standard deviations (SDs) below the mean normative values on at least 2 out of 6 cognitive domains assessed with MACFIMS will be classified as having CI. Participants who score 1 to 1.99 SDs below the mean normative values on at least 1 cognitive domain and/or at least 2 SDs below the mean normative values on 1 cognitive domain (not fulfilling the CI criteria) will be classified as having MCI. The remaining participants will be defined as CP [65]. For the Multiple Screener, participants scoring at least 2 SDs below the mean normative values on at least 1 of the 3 tests will be classified as CI. Participants scoring 1 to 1.99 SDs below the mean normative values on at least 1 of the 3 tests will be classified as MCI. The remaining participants will be defined as CP. Overall, regression-based norms adjusted for age, sex, and education will be used for individual cognitive tests before determining cognitive status.

Participants' cognitive status will be determined via the MACFIMS and will be investigated in relation to the scores detected with Multiple Screener^©^. All accuracy values will be calculated separately for the detection of MCI and CI (one against all approach for multiclass classification) and will be presented as percentages. Additionally, receiver-operating characteristic (ROC) analyses will be performed to determine overall accuracy and optimal cut-off scores of the Multiple Screener^©^ for detecting MCI and CI in people with MS. Once we have determined accuracy values and optimal cut-off scores in 300 participants, we will test these in another subset of at least 150 participants to confirm their correctness.

The Multiple Screener^©^ will be considered a sufficiently adequate screening instrument for the detection of (M)CI if its overall sensitivity values are at least 0.80 and specificity values at least 0.70. However, if one of the individual tests does not meet these criteria, we will determine accuracy values of the two other tests over and above that of all three tests combined.

#### Secondary study parameters

##### Test–retest reliability

Test–retest reliability of the Multiple Screener^©^ will be determined by calculating intraclass correlation coefficients (ICCs) for absolute agreement, using a two-way mixed model. Based on the 95% confidence interval of the ICC estimate, values will be considered to reflect poor reliability (< 0.5), moderate (0.5 -0.75), good (0.75–0.9), and excellent (> 0.90) [66]. The coefficients will be calculated separately for the SDMT, CVLT-II, and the SPART.

##### Relationships between cognition and psychological, work-related, and patient-reported health-related quality of life measures

Cross-sectional associations between cognition and psychological, work-related, and health-related quality of life measures will be analysed using Pearson’s or Point-Biserial correlations and linear regression analyses (including stepwise procedures) in both subsets and the overall sample. Correlations coefficients of less than 0.3, between 0.3 and 0.7, and greater than 0.7 will be considered weak, moderate, and strong, respectively [67]. An overview of the hypothesized correlations can be found in Table 4.

Additionally, logistic regression analyses will be used to identify the predictive value of demographical and disease characteristics (such as sex, MS subtype, medication, comorbidities) on cognitive functioning. Additionally, differences between groups (CP, MCI and CI) in demographic and clinical characteristics and other outcome measurements (e.g., psychological, work-related and health-related quality of life measures) will be analysed using independent samples t-tests, Mann–Whitney U tests and Pearson’s chi-square tests. For particular analyses, confounding variables (such as age, sex, education, EDSS score, disease duration, mood, fatigue etc.) will be inserted. Bonferroni corrections will be applied to correct for multiple comparisons within each objective.

### Safety reporting

We will not collect information on (serious) adverse events due to the observational and non-interventional nature of this study.

### Study status

The first participant was included on 19 July 2022. Currently 216 participants have been enrolled in the study (December 2023).

## Discussion

Cognitive impairment is common in PwMS and can severely affect health-related quality of life. In order to intervene timely, a baseline assessment and frequent monitoring of cognitive functioning seems crucial. However, in the Netherlands, neuropsychological assessment is not (yet) integrated into standard care due to the time-consuming nature of cognitive testing and limited availability of trained personnel [19]. The current study will validate a digital screening tool with the primary objective to enable early identification of cognitive decline in PwMS. The validation of the Multiple Screener^©^ within a representative sample of PwMS that visit a neurologist, will lay the foundation for implementing a cognitive screening tool for annual testing in clinical practice in the near future. This study will further help raise awareness among health care professionals about cognitive impairment in MS and its significance within the broader scheme of priorities in MS-care. The fact that 12 hospitals in the Netherlands are interested in participating in the study further emphasizes the need for such screening methods. In addition, this study will also contribute to the development of practical guidelines for Dutch professionals regarding the screening and subsequent monitoring of cognitive decline in MS.

Moreover, with the present study we are collecting one of the largest datasets on cognition and its determinants in PwMS which will provide us with a wealth of data that can be used to answer multiple relevant related research questions. Specifically, it will enhance our understanding of the relationship between cognition and relevant confounders, ranging from cognitive self-awareness to fatigue and mood problems.

To conclude, the validation of the Multiple Screener^©^ will facilitate early identification of cognitive impairment in PwMS; ultimately enabling better management of cognitive symptoms in this population. Additionally, the study's comprehensive dataset will allow new insights into factors related to cognition in PwMS, thus informing future research and clinical practices. Finally, timely identification of cognitive impairment is a crucial step for initiating early interventions, an important aspect that will be explored in subsequent phases of the larger *Don't be late!* study.

## Data Availability

Not applicable.

## Abbreviations

AIS: Athens Insomnia Scale
ASMT: Amsterdam Short Term Memory Test
BICAMS: Brief International Cognitive Assessment for MS
BVMS NL version: Buffalo Vocational Monitoring Survey
BVMT-R: Brief visuospatial memory test-revised
CD-RISC 25: Connor Davidson Resilience Scale
CI: Cognitively impaired
COWAT: Controlled oral word association test
CP: Cognitively preserved
CVLT: California verbal learning test
D-KEFS: Delis-Kaplan Executive Function System sorting test
EDSS: Expanded disability status scale
HADS: Hospital anxiety and depression scale
ICCs: Intraclass correlation coefficients
JLO: Judgment of line orientation test
LTE: List of Threatening Events Questionnaire
MACFIMS: Minimal Assessment of Cognitive Function in Multiple Sclerosis
MCI: Mild cognitive impairment
METC: Medical Ethics Committee
MFIS: Modified Fatigue Impact Scale
MS: Multiple Sclerosis
MSIS-29: Multiple Sclerosis Impact Scale
MSNQ: Multiple Sclerosis Neuropsychological Questionnaire
MSWDQ-23: Multiple Sclerosis Work Difficulties Questionnaire
NEO-FFI: NEO Five-Factor Inventory
PMS: Pearlin Mastery Scale
PwMS: People with Multiple Sclerosis
ROC: Receiver-operating characteristic
SDMT: Symbol digit modalities test
SF-36: 36-Item Short Form
SPART: Spatial Recall Test
WMO: Medical Research Involving Human Subjects Act
WPAI: Work Productivity and Activity Impairment Questionnaire

## References

[1] Katz Sand I (2015). Classification, diagnosis, and differential diagnosis of multiple sclerosis. Curr Opin Neurol.

[2] Campbell J, Rashid W, Cercignani M, Langdon D (2017). Cognitive impairment among patients with multiple sclerosis: associations with employment and quality of life. Postgrad Med J.

[3] Sumowski JF (2018). Cognition in multiple sclerosis: State of the field and priorities for the future. Neurology.

[4] Eijlers AJC (2018). Predicting cognitive decline in multiple sclerosis: a 5-year follow-up study. Brain.

[5] Wojcik C (2022). Staging and stratifying cognitive dysfunction in multiple sclerosis. Mult Scler.

[6] Benedict RH (2014). Negative work events and accommodations in employed multiple sclerosis patients. Mult Scler.

[7] Kordovski VM (2015). Identifying employed multiple sclerosis patients at-risk for job loss: when do negative work events pose a threat?. Mult Scler Relat Disord.

[8] van der Hiele K (2015). Work participation and executive abilities in patients with relapsing-remitting multiple sclerosis. PLoS ONE.

[9] van Gorp DAM (2019). Cognitive functioning as a predictor of employment status in relapsing-remitting multiple sclerosis: a 2-year longitudinal study. Neurol Sci.

[10] Global MS Employment Report 2016. 2016, MS International Federation: msif.org.

[11] Uitdehaag B (2017). New insights into the burden and costs of multiple sclerosis in Europe: results for the Netherlands. Multiple Sclerosis J.

[12] Meide HVD, Gorp DV, Van Der Hiele K, Visser L (2018). “Always looking for a new balance”: toward an understanding of what it takes to continue working while being diagnosed with relapsing-remitting multiple sclerosis. Disabil Rehabil.

[13] Pope C, Ziebland S, Mays N (2000). Qualitative research in health care. Analysing qualitative data. BMJ.

[14] Prouskas SE, et al. A randomized trial predicting response to cognitive rehabilitation in multiple sclerosis: Is there a window of opportunity? Mult Scler. 2022:13524585221103134. 10.1177/13524585221103134 PMC9574229 35765748

[15] Fuchs TA (2019). Response heterogeneity to home-based restorative cognitive rehabilitation in multiple sclerosis: an exploratory study. Mult Scler Relat Disord.

[16] Fuchs TA (2020). Functional connectivity and structural disruption in the default-mode network predicts cognitive rehabilitation outcomes in multiple sclerosis. J Neuroimaging.

[17] Taylor LA (2023). Understanding who benefits most from cognitive rehabilitation for multiple sclerosis: a secondary data analysis. Mult Scler.

[18] Kalb R (2018). Recommendations for cognitive screening and management in multiple sclerosis care. Mult Scler.

[19] Klein OA, das Nair R, Ablewhite J, Drummond A. Assessment and management of cognitive problems in people with multiple sclerosis: a National Survey of Clinical Practice. Int J Clin Pract. 2018:e13300. 10.1111/ijcp.13300 30507025

[20] van Dongen L (2019). Introducing multiple screener: an unsupervised digital screening tool for cognitive deficits in MS. Mult Scler Relat Disord.

[21] Langdon DW (2012). Recommendations for a Brief International Cognitive Assessment for Multiple Sclerosis (BICAMS). Mult Scler.

[22] Smith A. Symbol digit modalities test. 1973: Western Psychological Services Los Angeles.

[23] Delis D (1989). Neuropsychological assessment of learning and memory. Cortex.

[24] Woods SP (2006). The California Verbal Learning Test–second edition: Test-retest reliability, practice effects, and reliable change indices for the standard and alternate forms. Arch Clin Neuropsychol.

[25] Mulder JL, Dekker R, Dekker PH (1996). Verbale Leer en Geheugen Test.

[26] Rao SM. Cognitive Function Study Group, N. A Manual for the Brief Repeatable Battery of Neuropsychological Tests in Multiple Sclerosis., N.M.S. Society, Editor. 1990: New York.

[27] Zigmond AS, Snaith RP (1983). The hospital anxiety and depression scale. Acta Psychiatr Scand.

[28] Kos D (2003). Assessing fatigue in multiple sclerosis: Dutch modified fatigue impact scale. Acta Neurol Belg.

[29] Benedict RH (2003). Screening for multiple sclerosis cognitive impairment using a self-administered 15-item questionnaire. Mult Scler.

[30] Benedict RH (2006). Validity of the minimal assessment of cognitive function in multiple sclerosis (MACFIMS). J Int Neuropsychol Soc.

[31] Bossuyt PM (2015). STARD 2015: an updated list of essential items for reporting diagnostic accuracy studies. BMJ.

[32] Benedict RH (2012). Brief International Cognitive Assessment for MS (BICAMS): international standards for validation. BMC Neurol.

[33] Thompson AJ (2018). Diagnosis of multiple sclerosis: 2017 revisions of the McDonald criteria. Lancet Neurol.

[34] Kurtzke JF (1983). Rating neurologic impairment in multiple sclerosis: an expanded disability status scale (EDSS). Neurology.

[35] Benedict RH (1996). Revision of the Brief visuospatial memory test: studies of normal performance, reliability, and validity. Psychol Assess.

[36] Gronwall D (1977). Paced auditory serial-addition task: a measure of recovery from concussion. Percept Mot Skills.

[37] Benton AL, Sivan AB, Hamsher K, Varney NR, Spreen O (1994). Contributions to Neuropsychological Assessment.

[38] Delis DC, Kaplan E, Kramer JH (2001). Delis-Kaplan executive function system.

[39] Schagen S, Schmand B, de Sterke S, Lindeboom J (1997). Amsterdam Short-Term Memory test: a new procedure for the detection of feigned memory deficits. J Clin Exp Neuropsychol.

[40] Nauta IM et al. Performance validity in outpatients with multiple sclerosis and cognitive complaints. Mult Scler. 2021:13524585211025780. 10.1177/13524585211025780 PMC8961248 34212754

[41] Lee GP, Loring DW, Martin RC. Rey’s 15-item visual memory test for the detection of malingering: normative observations on patients with neurological disorders. Psychol Assess. 1992;4(1):43–6.

[42] Baron-Cohen S (2001). The “Reading the Mind in the Eyes” Test revised version: a study with normal adults, and adults with Asperger syndrome or high-functioning autism. J Child Psychol Psychiatry Allied Disciplines.

[43] Rothlind J, Dukarm P, Kraybill M (2017). Assessment of self-awareness of cognitive function: correlations of self-ratings with actual performance ranks for tests of processing speed, memory and executive function in non-clinical samples. Arch Clin Neuropsychol.

[44] Patel AA, Donegan D, Albert T (2007). The 36-item short form. J Am Acad Orthop Surg.

[45] Hobart J (2001). The multiple sclerosis impact scale (MSIS-29) a new patient-based outcome measure. Brain.

[46] Soldatos CR, Dikeos DG, Paparrigopoulos TJ (2000). Athens Insomnia Scale: validation of an instrument based on ICD-10 criteria. J Psychosom Res.

[47] Soldatos CR, Dikeos DG, Paparrigopoulos TJ (2003). The diagnostic validity of the Athens Insomnia Scale. J Psychosom Res.

[48] Connor KM (2006). Assessment of resilience in the aftermath of trauma. J Clin Psychiatry.

[49] Pearlin LI, Schooler C. The structure of coping. J Health Soc Behav. 1978:2–21. 649936

[50] Costa Jr PT, McCrae RR. The Revised Neo Personality Inventory (neo-pi-r). 2008: Sage Publications, Inc.

[51] Schwartz ES (2011). The NEO-FFI in multiple sclerosis: internal consistency, factorial validity, and correspondence between self and informant reports. Assessment.

[52] Donnellan MB, Oswald FL, Baird BM, Lucas RE (2006). The mini-IPIP scales: tiny-yet-effective measures of the Big Five factors of personality. Psychol Assess.

[53] Brugha TS, Cragg D (1990). The list of threatening experiences: the reliability and validity of a brief life events questionnaire. Acta Psychiatr Scand.

[54] Reilly MC, Zbrozek AS, Dukes EM (1993). The validity and reproducibility of a work productivity and activity impairment instrument. Pharmacoeconomics.

[55] Honan CA, Brown RF, Hine DW (2014). The multiple sclerosis work difficulties questionnaire (MSWDQ): development of a shortened scale. Disabil Rehabil.

[56] van Egmond E et al. A Dutch validation study of the Multiple Sclerosis Work Difficulties Questionnaire in relapsing remitting multiple sclerosis. Disabil Rehabil. 2019:1–10. 10.1080/09638288.2019.1686072 31702954

[57] Benedict RH (2014). Negative work events and accommodations in employed multiple sclerosis patients. Mult Scler J.

[58] Foley T, McKinlay A, Warren N, Stolwyk RJ. Assessing the sensitivity and specificity of cognitive screening measures for people with Parkinson’s disease. NeuroRehabilitation. 2018;43(4):491–500. 10.3233/NRE-182433 30400110

[59] Dusankova JB, Kalincik T, Havrdova E, Benedict RH (2012). Cross cultural validation of the Minimal Assessment of Cognitive Function in Multiple Sclerosis (MACFIMS) and the Brief International Cognitive Assessment for Multiple Sclerosis (BICAMS). Clin Neuropsychol.

[60] Amato MP, Ponziani G, Siracusa G, Sorbi S (2001). Cognitive dysfunction in early-onset multiple sclerosis: a reappraisal after 10 years. Arch Neurol.

[61] Bujang M, Adnan TH (2016). Requirements for minimum sample size for sensitivity and specificity analysis. J Clin Diagn Res.

[62] Aarts J, Saddal SRD, Bosmans JE, de Groot V, de Jong BA, et al. Don’t be late! Postponing cognitive decline and preventing early unemployment in people with multiple sclerosis: a study protocol. BMC Neurology. 2024. 10.1186/s12883-023-03513-y. 10.1186/s12883-023-03513-y PMC10789039 38225561

[63] RStudio (2020). TeamRStudio: Integrated Development for R. RStudio.

[64] Corp I (2021). IBM SPSS Statistics for Windows, Version 28.0.

[65] Eijlers AJ (2017). Increased default-mode network centrality in cognitively impaired multiple sclerosis patients. Neurology.

[66] Koo TK, Li MY (2016). A Guideline of selecting and reporting intraclass correlation coefficients for reliability research. J Chiropr Med.

[67] Akoglu H. User’s guide to correlation coefficients. Turk J Emerg Med. 2018;18(3):91–3. 10.1016/j.tjem.2018.08.001 PMC6107969 30191186

